# Prevention of Surgical Site Infections in Gynecologic Surgery: A Review of Risk Factors and Recommendations

**DOI:** 10.31486/toj.20.0044

**Published:** 2020

**Authors:** Veronica Gillispie-Bell

**Affiliations:** Department of Obstetrics and Gynecology, Ochsner Clinic Foundation, New Orleans, LA and The University of Queensland Faculty of Medicine, Ochsner Clinical School, New Orleans, LA

**Keywords:** *Gynecologic surgical procedures*, *quality improvement*, *risk factors*, *surgical wound infection*

## Abstract

**Background:** Surgical site infections (SSIs) are a type of health care–associated infection that can cause significant patient harm. Many are preventable. Postoperative courses complicated by an SSI can equate to longer hospital stays, lost time from work, and the need for reoperation.

**Methods:** This review addresses types of SSIs, risk factors, and best practices for preventing SSIs associated with gynecologic surgery.

**Results:** Best practices to reduce SSIs are divided into preoperative, intraoperative, and postoperative activities. Preoperative considerations include patient showering, hair removal, glycemic control, and hand and forearm scrub. Intraoperative concerns are antibiotic prophylaxis, skin preparation prior to the start of surgery, and the operating room environment. Postoperative concerns are surgical dressing, vacuum-assisted wound closure, and patient instructions.

**Conclusion:** Best practices should be established and followed to reduce the risk of SSI associated with gynecologic surgery.

## INTRODUCTION

Surgical site infections (SSIs) are a type of health care–associated infection that can cause significant patient harm. Postoperative courses complicated by an SSI can equate to longer hospital stays, lost time from work, and the need for reoperation. Data from 2009 demonstrate that hospital stays were extended an average of 9.7 days because of SSIs, with an average cost of $20,842 per hospital stay.^1^ A study conducted at The Johns Hopkins Hospital during a 3-year period found an SSI rate of 2.76 per 100 surgical procedures, resulting in a net loss in profit between $4,147 and $22,239 per SSI.^2^ According to the Centers for Disease Control and Prevention (CDC), the mortality rate associated with SSI is 3%.^3^ However, with the use of best practices, 40% to 60% of SSIs are preventable.^4^ The cost to hospital facilities combined with the high preventability has led many hospital systems to use SSI rate as a value-based metric.

## CLASSIFYING A SURGICAL SITE INFECTION

The CDC and the National Healthcare Safety Network have clear definitions for the 3 categories of SSIs that occur within 30 days after a procedure: superficial, deep, and organ space (Table 1).^3^ A superficial SSI is confined to the skin or subcutaneous tissue, a deep SSI involves deep soft tissue, and an organ space SSI involves a body part that is below the fascia.

**Table 1.** Categories of Surgical Site Infections^3^

Category	Affected Tissue	Characteristics
Superficial	Superficial tissue, skin, and subcutaneous tissue	One of the following required: Purulent drainage Organism appropriately obtained through wound culture Incision opened by physician, surgeon, or advanced practice practitioner and presence of pain, swelling, warmth, or redness Diagnosed as a superficial surgical site infection by a physician, surgeon, or advanced practice practitioner
Deep	Deep tissue, fascia, or muscle	One of the following required: Purulent drainage Spontaneous dehiscence or incision opened by a surgeon, organism appropriately obtained through wound culture, and presence of pain or fever Abscess identified by examination, surgery, or imaging
Organ space	Below the fascia and muscle	One of the following required: Purulent drainage from a drain in the deep organ space Organism appropriately obtained through wound culture Abscess identified by examination, surgery, or imaging

## RISK FACTORS

Risk factors for SSIs can be divided into 2 groups: patient risk factors and operative risk factors (Table 2).

**Table 2.** Risk Factors for Surgical Site Infections

Patient Risk Factors	Operative Risk Factors
Smoking Diabetes Obesity Malnutrition Anticoagulation Presence of infection Age	Length of surgery Blood transfusion Wound classification (clean, clean-contaminated, contaminated, dirty/infected)

### Patient Risk Factors

According to the American College of Surgeons, patients who are active smokers have a 40% higher risk of postoperative surgical complications, including SSI,^5^ because of vasoconstriction that leads to tissue hypoxia. Additionally, smoking alters the immune response. Because of the significant risk for postoperative complication, the American College of Surgeons recommends that patients stop smoking for at least 4 to 6 weeks prior to surgery.

Obesity is also an independent risk factor for developing an SSI, particularly for patients undergoing abdominal surgeries such as hysterectomy.^6^ The pathophysiology of obesity and the increased risk of SSI is thought to be attributed to the increased ratio of adipose tissue to capillary density, which leads to poor tissue perfusion. Because of the increased risk of SSI with obesity, appropriate weight-based preoperative antibiotics should be administered to reduce the risk.

### Operative Risk Factors

Longer surgery duration, even with appropriate redosing of antibiotics, has been identified as an independent risk factor for developing an SSI.^7^ Intraoperative blood transfusion also increases the risk of SSI, especially for organ space infections.^8^ Wound classification is another operative risk factor. The CDC classifies operative procedures and their wounds as clean, clean-contaminated, contaminated, or dirty/infected (Table 3).^3^ Hysterectomy is classified as a clean-contaminated procedure. In general, clean-contaminated procedures have an infection rate of 3.94%; the aim of best practices is to lower this rate.^9^

**Table 3.** Wound Classification and Infection Risk^3,9^

Wound Classification	Description	Risk of Surgical Site Infection, %
Clean	Uninfected operative wound with no inflammation Does not involve respiratory, alimentary, genital, or urinary tract	1.76
Clean-contaminated	Operative wound involving the respiratory, alimentary, genital, or urinary tract	3.94
Contaminated	Open, fresh, accidental wound Major breaks in sterile technique Gross spillage from the gastrointestinal tract Nonpurulent inflammation including necrotic tissue	4.75
Dirty/infected	Old, traumatic wounds with retained devitalized tissue Clinical infection or perforated viscera	5.16

## BEST PRACTICES TO REDUCE SURGICAL SITE INFECTION

Best practices to reduce SSIs are divided into preoperative, intraoperative, and postoperative activities.

### Preoperative Considerations

#### Patient Showering

Patients should shower with soap or an antiseptic agent at least the night before surgery. While preoperative showering has been shown to reduce the rate of SSIs, a 2015 Cochrane review demonstrated no benefit to showering with bar soap vs chlorhexidine.^10^ However, the data showed a statistically significant reduction in SSIs after a full wash with chlorhexidine vs a partial wash. Of note, the method by which the wash was performed was not standardized in the studies included in the Cochrane review. Another study published in the *Journal of the American Medical Association* showed a reduction in SSIs when the wash was standardized: a minimum of 2 sequential showers and a 1-minute pause before rinsing.^11^ Given the variations in studies and the lack of conclusive data, the literature does not provide consensus on how the shower should be performed or what type of cleanser should be used.

#### Hair Removal

Patients should be instructed to not remove hair at home prior to surgery. For surgical purposes, hair should not be removed unless it will interfere with the procedure. If hair needs to be removed, clippers instead of a razor should be used because razors can cause microtrauma to the skin that can be a nidus of infection. Preoperative nurses should be instructed to make the hair lower but not to make the area bald, because making an area bald can also cause microtrauma to the skin. Hair should be removed in the preoperative area and not the operating room.^1^

#### Glycemic Control

The stress of surgery causes dysregulation in glucose production and glucose utilization, thereby increasing the risk of SSI. From 12% to 30% of patients undergoing surgery are found to have hyperglycemia, even in the absence of a history of diabetes.^12^ Therefore, performing a fasting blood sugar test on all patients prior to surgery, regardless of their history of diabetes, is important. The target glucose level is debatable. The Society for Ambulatory Anesthesia,^13^ the American Diabetes Association/American Association of Clinical Endocrinologists,^14^ the Endocrine Society,^15^ and the Society of Thoracic Surgeons^16^ all recommend a glucose level <180 mg/dL, while the CDC recommends preoperative glucose <200 mg/dL to reduce the risk of SSI.^17^ Based on data from critically ill patients in intensive care units, glucose <110 mg/dL is associated with adverse outcomes and should be avoided as well.^18^

#### Hand and Forearm Scrub

The traditional 10-minute hand and forearm scrub is no longer recommended. Scrubbing for 2 to 6 minutes is just as effective for reducing bacteria without the skin damage that can result from the 10-minute scrub.^19^ Either an antimicrobial soap or an alcohol-based scrub should be used with or without a sponge but not with a brush. Alcohol-based scrubs provide an immediate antimicrobial effect because of the denaturation of proteins and are effective against most gram-positive and gram-negative bacteria, including multidrug-resistant pathogens, but they do not provide persistent antimicrobial effect (approximately 1 to 3 hours of effect).^19^ Chlorhexidine gluconate causes disruption of cytoplasmic membranes, is more effective against gram-positive than gram-negative bacteria, and is more effective than alcohol-based solutions. Although not as immediately effective as alcohol-based solutions, chlorhexidine gluconate lasts for at least 6 hours.^19^ Iodophor/iodine scrubs cause impaired protein synthesis and alteration of cell membranes, provide rapid onset of action, and are effective against gram-positive and gram-negative bacteria.^19^ Alcohol-based scrubs with chlorhexidine provide the best immediate and persistent antimicrobial activity. A prewash with a nonantimicrobial soap and drying before applying the alcohol-based scrub is recommended. Even with appropriate washing, all skin flora and bacteria may not be removed. Additionally, bacteria reaccumulate over time—an especially important consideration during lengthy procedures—so double-gloving is recommended.

### Intraoperative Considerations

#### Antibiotic Prophylaxis

Appropriate weight-based antibiotics should be administered prior to the start of surgery. According to the Clinical Practice Guidelines for Antimicrobial Prophylaxis in Surgery, cefazolin is a first-line recommended prophylactic antibiotic.^20^ The recommended dose is 2 grams for patients weighing <120 kg and 3 grams for patients ≥120 kg, administered up to 30 minutes prior to incision.^20^ For patients with a severe allergy to cefazolin, clindamycin or vancomycin plus an aminoglycoside, such as gentamicin, is recommended and can be administered up to 2 hours prior to incision.^20^ Alternatively, metronidazole plus an aminoglycoside or fluoroquinolone can be used. In addition to administering the appropriate antibiotics prior to surgery, care must be taken to readminister antibiotics when necessary. Because of the half-life of cefazolin, readministration is recommended 4 hours after the initial dose. The redosing interval for clindamycin is 6 hours. Redosing of gentamicin and vancomycin is not recommended. Redosing of antibiotics is also recommended in cases of excessive blood loss, defined as ≥1,500 mL.

#### Skin Preparation in the Operating Room

Prior to making the surgical incision, the skin is cleaned to remove microorganisms. Several skin preparations are available, and they vary in onset of action, duration, and antimicrobial coverage (Table 4).^21^ Based on these factors, a solution of alcohol with chlorhexidine gluconate is preferred, such as ChloraPrep (2% chlorhexidine gluconate and 70% isopropyl alcohol). ChloraPrep must be applied appropriately to ensure effectiveness and safety.^22^ The skin must be prepped for 30 seconds in dry areas and for 2 minutes in moist areas, such as underneath a pannus. To prevent fires in the operating room, the solution must be allowed to dry. In hairless areas, the recommended drying time is 3 minutes. In areas with hair, the recommended drying time is 1 hour.

**Table 4.** Antiseptic Skin Preparations^21^

Antiseptic	Mechanism of Action	Antimicrobial Coverage	Onset	Application	Duration	Examples
Aqueous-iodophor	Causes protein damage and DNA damage	Gram positive, gram negative, fungi, viruses	Intermediate	2-step scrub and paint	2 h	Betadine Scrub Care
Aqueous-CHG	Disrupts membrane	Gram positive, gram negative, fungi, viruses	Intermediate	2-step scrub and dry, repeat	6 h	Hibiclens
Alcohol-iodophor	Denatures protein and causes DNA damage	Gram negative	Rapid	1-step paint, dry time of 3 min in hairless areas	48 h	DuraPrep
					96 h	Prevail-FX
Alcohol-CHG	Denatures protein and disrupts membrane	Gram negative	Rapid	30 s or 2 min, dry time of 3 min in hairless areas	48 h	ChloraPrep

CHG, chlorhexidine gluconate.

#### Operating Room Environment

Perioperative hypothermia can increase the risk of SSI. Kurz et al found a 3-times increased risk of SSI in patients who became hypothermic (34.5 °C/94.1 °F) during elective colorectal resection for cancer or inflammatory bowel disease.^23^ This increased risk is attributable to decreased blood perfusion that decreases antibiotic penetration into the subcutaneous and adipose tissue. Hypothermia also increases blood loss, decreases wound healing, and increases cardiac morbidity.^24,25^ Normothermia is defined as a core temperature of at least 36 °C on arrival to the postanesthesia care unit.^26^

### Postoperative Considerations

#### Surgical Site Dressing

A 2016 Cochrane review demonstrated that no one dressing was superior for prevention of SSI,^27^ so consensus is lacking on what type of dressing is best for prevention of SSI. However, the CDC recommends that the dressing remain in place for 24 to 48 hours after surgery.^28^

#### Vacuum-Assisted Wound Closure

Prophylactic vacuum-assisted wound closure has been shown to reduce the risk of SSI in patients who undergo cesarean section*.*^29^ In a 2019 Cochrane review, using a negative pressure vacuum for primary wound closure was associated with a decreased incidence of SSI compared to routine dressing in the general, orthopedic, and obstetric surgical units in acute care hospitals.^30^ Although the review was not specific to gynecologic surgery, the results demonstrated in other types of surgery are promising. More studies are needed to determine if prophylactic vacuum application is a cost-effective means of reducing SSIs in gynecologic surgeries with abdominal incisions.

#### Patient Instructions

Patients need to be engaged in preventing SSIs. A review conducted by Tartari et al, based on current recommendations and an expert panel, demonstrated that patient instructions should address hair removal, smoking cessation, preoperative showering, and wound care after surgery.^31^ Postoperative instructions must be appropriate to the health literacy of the patients receiving them.^32^

## SURGICAL BUNDLES

Implementation of surgical care bundles has been shown to reduce SSI rates in colorectal surgery,^33^ orthopedic surgery,^34^ and spinal surgery.^35^ The Council on Patient Safety in Women's Health Care created a bundle to prevent SSIs specific to gynecologic surgery.^36^ The prevention of surgical site infections after major gynecologic surgery bundle is divided into 4 sections: readiness, recognition and prevention, response, and reporting and systems learning. The readiness section calls for all facilities to implement preoperative and intraoperative best practices as well as a team approach to preventing SSIs. The recognition section addresses assessment of modifiable and nonmodifiable patient risk factors for every patient. The response section suggests intraoperative timeouts to address patient-specific issues, as well as reassessment of patient risk. The reporting and systems learning section focuses on collecting, analyzing, and sharing data. To achieve success, however, quality improvement techniques and tools are needed to augment the best practice recommendations of the bundle. The bundle, combined with improvement in science, can improve SSI rates.

## CONCLUSION

Some risk factors for SSI are modifiable, and best practices should be established and followed to reduce those risks. While several preoperative, intraoperative, and postoperative best practices have been demonstrated to reduce the risk of SSIs, research is still needed to determine best practices for some aspects of the surgical process. Using a bundled approach in conjunction with quality improvement tools can make a positive impact in the reduction of SSIs.

## Notes

This article meets the Accreditation Council for Graduate Medical Education and the American Board of Medical Specialties Maintenance of Certification competencies for Patient Care and Medical Knowledge.
